# Monte Carlo Method with Heuristic Adjustment for Irregularly Shaped Food Product Volume Measurement

**DOI:** 10.1155/2014/683048

**Published:** 2014-05-05

**Authors:** Joko Siswantoro, Anton Satria Prabuwono, Azizi Abdullah, Bahari Idrus

**Affiliations:** ^1^Faculty of Information Science and Technology, Universiti Kebangsaan Malaysia (UKM), 43600 Bangi, Selangor Darul Ehsan, Malaysia; ^2^Faculty of Engineering, University of Surabaya, Jl. Kali Rungkut, Surabaya 60293, Indonesia; ^3^Faculty of Computing and Information Technology, King Abdulaziz University, P.O. Box 344, Rabigh 21911, Saudi Arabia

## Abstract

Volume measurement plays an important role in the production and processing of food products. Various methods have been proposed to measure the volume of food products with irregular shapes based on 3D reconstruction. However, 3D reconstruction comes with a high-priced computational cost. Furthermore, some of the volume measurement methods based on 3D reconstruction have a low accuracy. Another method for measuring volume of objects uses Monte Carlo method. Monte Carlo method performs volume measurements using random points. Monte Carlo method only requires information regarding whether random points fall inside or outside an object and does not require a 3D reconstruction. This paper proposes volume measurement using a computer vision system for irregularly shaped food products without 3D reconstruction based on Monte Carlo method with heuristic adjustment. Five images of food product were captured using five cameras and processed to produce binary images. Monte Carlo integration with heuristic adjustment was performed to measure the volume based on the information extracted from binary images. The experimental results show that the proposed method provided high accuracy and precision compared to the water displacement method. In addition, the proposed method is more accurate and faster than the space carving method.

## 1. Introduction

There are several factors that must be considered in assessing the quality of food products in the food industry. One of these factors is the size of a food product. Size can be assessed by one-dimensional (1D) measurement, such as length, width, or Feret's diameter; by two-dimensional (2D) measurement, such as area and perimeter; and by three-dimensional (3D) measurement, such as volume and surface area [1]. Although 3D size measurement is more difficult, it plays a very important role in the production and processing of food products. Volume plays an important role in the sorting, grading [2, 3], and monitoring of fruit growth [4], as well as in determining other physical properties [5]. Therefore, research into 3D size measurement remains challenging, especially research into volume measurement.

The water displacement method based on Archimedes' principle has traditionally been used to measure the volume of food product. The product is submerged into the water, and the displaced water is considered as the volume of the product. This method is time consuming and inaccurate. For example, for porous objects, the water displacement method is lower in accuracy because the object will absorb water. Furthermore, for fragile objects, the water displacement method is considered a destructive method [5, 6]. Computer vision offers an alternative that is accurate, precise, and nondestructive for the size measurement of food products. A great deal of research has been performed to measure the size of food products using computer vision systems. A part of this research has used 2D computer vision, while others have used 3D computer vision [7]. 2D computer vision systems use one or two cameras to acquire one or two images of a measured object. The acquired images are then analyzed to measure the size of the object, including its diameter, perimeter, and projected area. 2D computer vision systems are also used to measure the volume of food products by assuming the products are ellipsoids or axisymmetric objects [4, 5, 8–10]. However, due to ellipsoid or axisymmetric object assumptions, 2D computer vision cannot be applied to measure the volume of food products with irregular shapes.

Generally, 3D computer vision systems use single or multiple cameras to capture multiple images of an object from different viewpoints. The images of such an object are used to reconstruct the 3D object, and the volume of object is measured from reconstructed 3D objects by mathematical formulations or by counting the number of voxels in reconstructed object. Lee et al. [3] proposed a nondestructive method of measuring the volumes and surface areas of an irregularly shaped object using a computer vision system. A 3D wireframe model for such an object was constructed based on a radial projection from images of the object taken by a single camera from at least 30 views at a fixed angular interval. The volume and surface area of the object were calculated from the 3D wireframe model using mathematical formulation. Goñi et al. [11] proposed a method to measure the volume and surface area of irregular foodstuffs using 3D reconstructions based on reverse engineering techniques. The foodstuffs were sliced along a selected axis and captured slice by slice to obtain the cross-sectional images of foodstuffs using a camera. The number of cross sections depends on the irregularity of the foodstuff and can range from 10 to 18. The images are then processed to make 3D solid objects. The volume and surface area of foodstuffs were calculated using finite element methods from reconstructed foodstuffs. Castillo-Castaneda and Turchiuli [6] presented a methodology to calculate the volume of milk-agglomerated particles. They used the same method as Lee et al. [3] for image acquisition. The 3D object was reconstructed from multiple views using the volume intersection method. The volume of the object was calculated from the 3D reconstructed object by counting the number of voxels. Although the volume measurement methods proposed by Lee et al. [3], Goñi et al. [11], and Castillo-Castaneda and Turchiuli [6] give highly accurate results, their computational costs are expensive. Furthermore, the method proposed by Goñi et al. [11] is considered to be destructive because the measured object should be sliced for image acquisition. Chalidabhongse et al. [2] developed the computer vision system to measure the size of mangoes, including their length, width, thickness, projected area, volume, and surface area. Space carving was employed to reconstruct 3D objects from four images of mangoes acquired by four cameras. The 1D and 2D sizes were measured from top view images, whereas volume and surface area were measured from reconstructed 3D objects by counting the number of voxels. Although the developed system employed four cameras to capture four images together, space carving used in 3D reconstruction is time consuming and produces high absolute relative error ranging from 8.89% to 9.96%.

Mathematically, the volume of a solid object enclosed by curved surfaces can be obtained by a triple integral over a region with boundaries at the surfaces of an object [12]. If the surface's equations for the object are known, then the integral can be evaluated either by analytical or numerical approximations. Otherwise, the surfaces of object must be reconstructed first, as in a 3D computer vision, for volume measurement. In such conditions, the integral can be evaluated by numerical approximation alone. An alternative method used to evaluate the integral is the Monte Carlo method. The Monte Carlo method is a numerical method that uses random number to solve mathematical problems [13]. It is a computationally effective method compared with deterministic methods in solving multidimensional problems and has been widely used to solve both mathematical and real problems, such as multidimensional integrals, linear equations, nonlinear equations, eigenvalue problems, boundary value problems, integral equations, path integrals, operations research, transport modeling in semiconductors and nanowires, radiation transport, statistical physics and chemistry, nuclear physics, traffic pattern modeling, and economics [14, 15]. The Monte Carlo method evaluates integrals by generating random points and using the arithmetic mean of the integrand, as evaluated at the generated random points, as an approximation [14]. Jaekel [16] presented the Monte Carlo method for high-dimensional volume estimation and application in polytopes. The vector integral approach was used in a high-dimensional volume estimation and evaluated using the Markov Chain Monte Carlo (MCMC) method. However, this approach was not implemented to measure the volume of a real object with an unknown surfaces equation.

Previous works show that the volume measurement of food products with irregular shapes was performed by 3D reconstruction. However, 3D object reconstruction makes the volume measurement process time consuming due to its computational cost. Furthermore, some of volume measurement methods based on 3D reconstruction have a low accuracy. Therefore, there is a need to develop an accurate and fast method to measure the volume of food product with irregular shapes. The objective of this paper was to propose a nondestructive volume measurement method using a computer vision system for food products with irregular shapes, based on the Monte Carlo method. To obtain the volume of an object, the Monte Carlo method needs only the information of whether certain generated random points fall inside or outside the object, without needing to know the surface of object. Therefore, the Monte Carlo method does not require 3D reconstruction to measure the volume of irregularly shaped objects.

## 2. Materials and Methods

### 2.1. Proposed Computer Vision System

**Figure 1:**
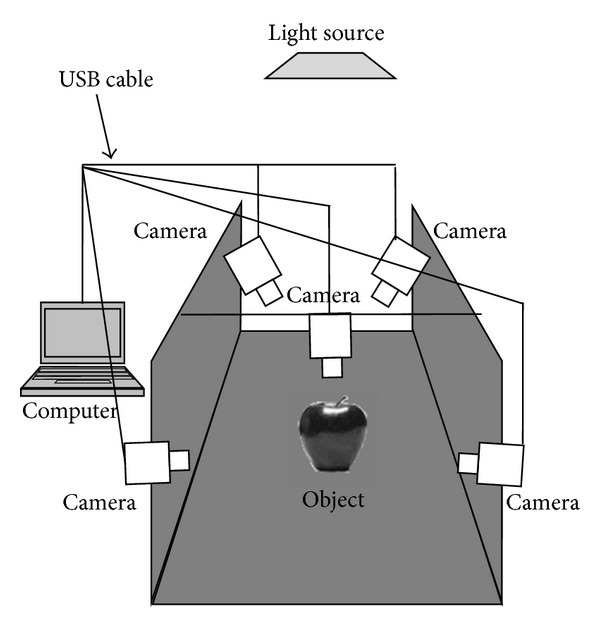
The experimental setup for the computer vision system.

**Figure 2:**
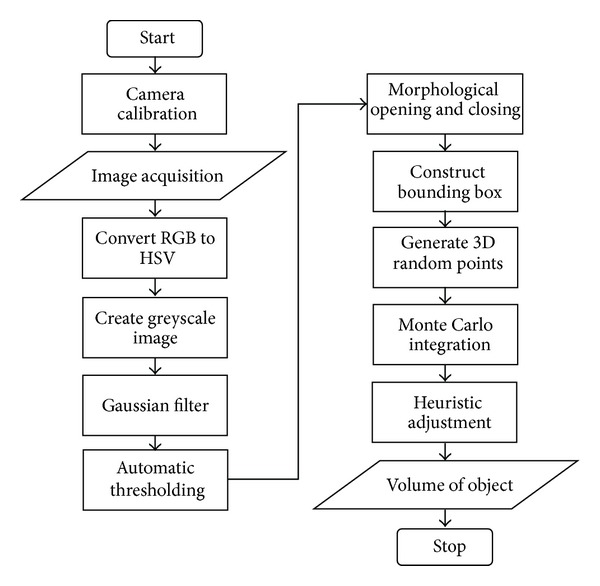
The flowchart of the proposed volume measurement method.

The computer vision system consists of hardware and software for camera calibration, image acquisition, image processing, and volume measurement based on the Monte Carlo method. The hardware for the computer vision system used in volume measurement included multiple cameras, a computer, a light source, and a black background, as shown in Figure 1. Five Logitech web cameras (one HD Pro Webcam C910 and four HD Webcam 270 h) were used for image acquisition. These cameras were connected to the computer using a USB cable. A 2.20 GHz Intel Core 2 Duo portable computer with Windows 7 operating system was used for the image processing and volume measurement. The light (tube lamp light) located on the ceiling of room was used as the light source. This type of light source was chosen to reduce the reflection of light onto the camera lens. The images of the measured object were acquired using a black background to obtain maximum contrast between the object and the background. With this condition, image segmentation can be easily performed. The main processing steps for the proposed volume measurement method consisted of camera calibration, image acquisition, preprocessing, segmentation, and volume measurement. The flowchart of these processing steps for the proposed method is depicted in Figure 2. The proposed method was implemented in Visual C++ 2010 using Open Source Computer Vision Library OpenCV 231 [17]. A user-friendly graphical user interface (GUI) was developed to interface between the computer vision system and the user.

### 2.2. Camera Calibration

The first step in the 3D computer vision system was camera calibration. The objective of the camera calibration was to obtain extrinsic and intrinsic camera parameters from 2D images. Extrinsic and intrinsic camera parameters are used to transform the coordinates of a point in a real world coordinate system to an image coordinate system. The proposed method employed a calibration technique based on Zhang's method [18]. A flat chessboard pattern with 9 × 6 corners and measuring 2.54 mm × 2.54 mm in each square was used as the calibration object.

Fifty-four inner corners in a chessboard pattern were used as points in a real world coordinate system and related to their coordinates in an image coordinate system to estimate the camera's parameters. Ten different views of the calibration object were used to estimate the intrinsic camera parameters. For the extrinsic camera parameter estimation, the calibration object was in the bottom of the measured object and was assumed to lie on plane *z* = 0 in the real world coordinate system. The center of the real world coordinate system was assumed to be in the top left corner of the chessboard pattern. Positive* x*- and* y*-axes were assumed to lie along the top left to bottom left corner and along the top left to top right corner, respectively. The positive* z*-axis was perpendicular to the* x*- and* y*-axes, according to the right hand rule. The results of camera calibration were an intrinsic matrix (containing the focal length in the* x* and* y* directions and the center of the image plane), rotation matrix, and translation vector. Because all cameras had a small distortion in both the radial and tangential distortions, the distortion matrix could be neglected.

### 2.3. Image Acquisition

**Figure 3:**
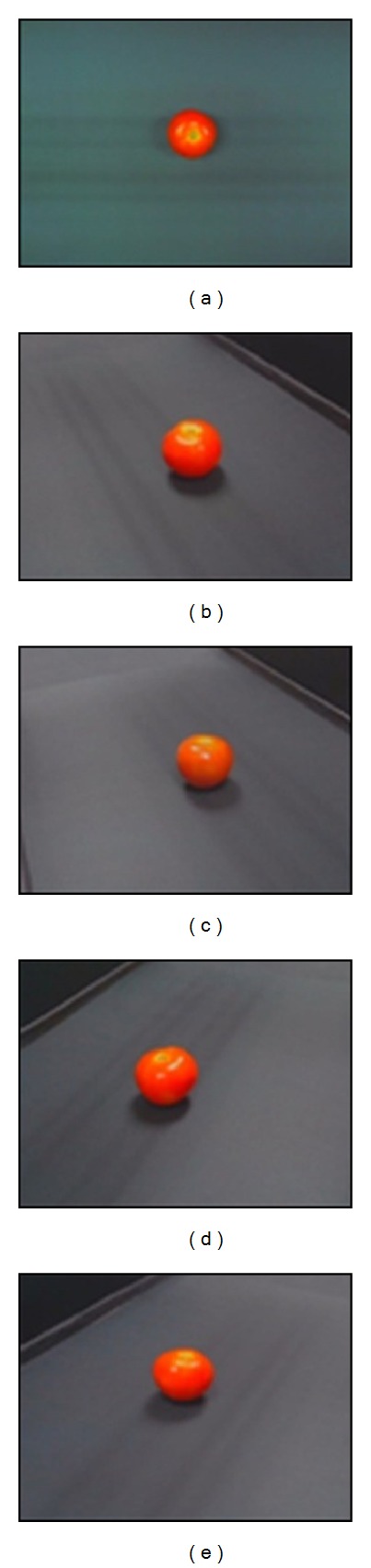
The samples of the captured images: (a) from the top view, (b)–(e) from the surrounding views.

The measured object was located at the center of a computer vision system and was assumed to lie on plane *z* = 0, as shown in Figure 1. Five images of the measured object were acquired using five cameras: one from the top view and four from the surrounding views. The images were captured in the RGB color space with a dimension of 640 × 480 pixels and a resolution of 96 dpi in both the vertical and horizontal directions. The samples of the captured image are shown in Figure 3.

### 2.4. Preprocessing

**Figure 4:**
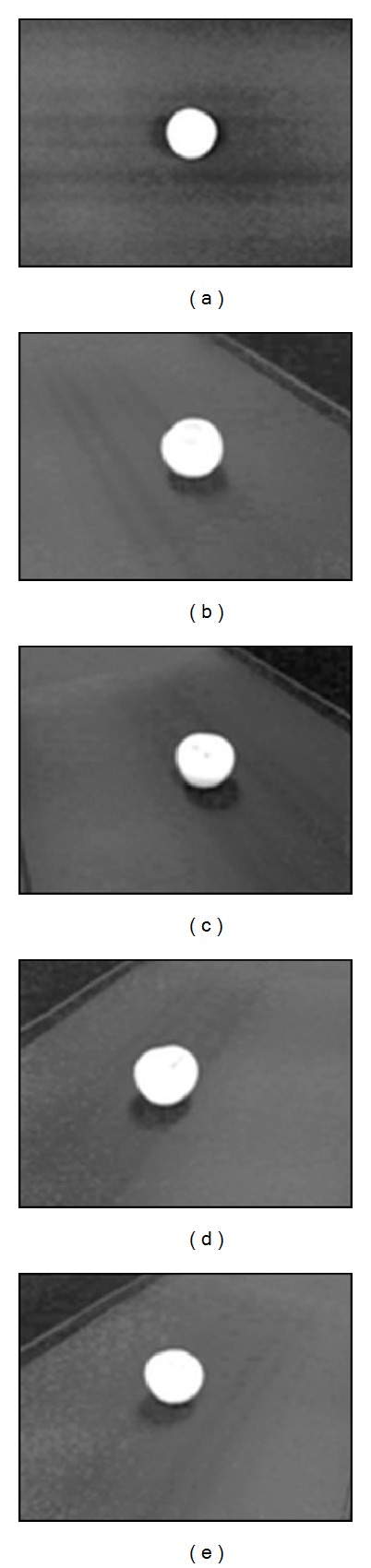
The samples of preprocessing result: (a) image from the top view, (b)–(e) images from the surrounding views.

To simplify the image segmentation process, the images of the measured object were converted from an RGB color space into a HSV color space. Because the measured objects could have a widely spread range of color, image segmentation could be easily performed in the HSV color space. In the H, S, or V component, the object could be easily separated from its background. A grayscale image was constructed from the weighted sum of H, S, and V components using

(1) $$\begin{array}{c} \text{Gr}=w_{h}\text{H}+w_{s}\text{S}+w_{v}\text{V}, \end{array}$$

where Gr is the grayscale image and *w*_*h*_, *w*_*s*_, *w*_*v*_ ∈ [0,1] are weights for the H, S, and V components, respectively. The values of the weights were chosen such that the optimum segmentation result would be obtained. Gr was then normalized to range [0, 255]. To enhance image quality through noise reduction, a 3 × 3 Gaussian filter was applied to the grayscale image.  Figure 4 shows the samples of the preprocessing results.

### 2.5. Image Segmentation

**Figure 5:**
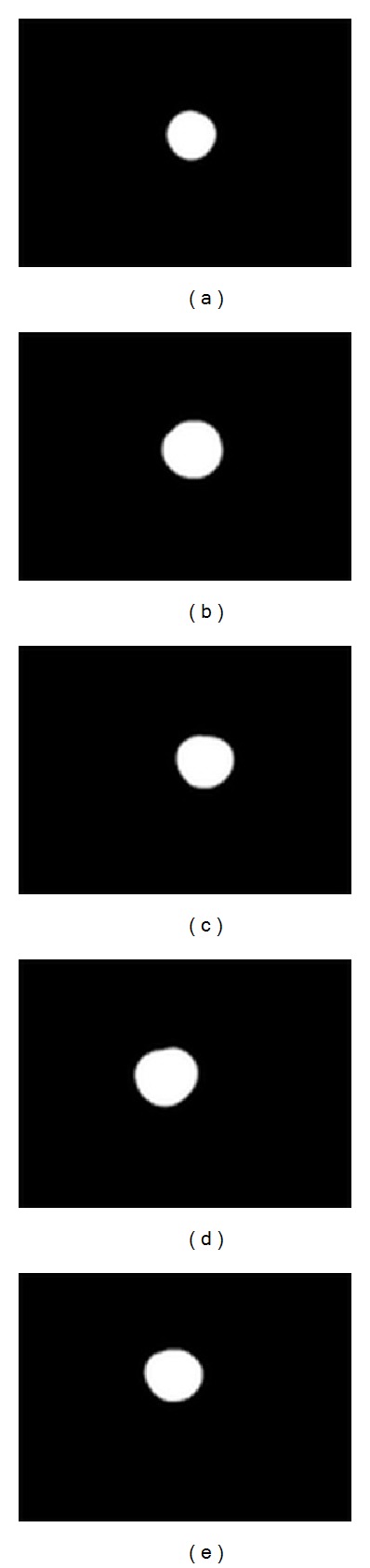
The samples of binary images: (a) image from top view, (b)–(e) images from surrounding views.

Segmentation decomposes an image into areas of interest and a background. The result of this step is called a binary image. The proposed method used thresholding to achieve image segmentation. An iterative procedure described by Gonzalez and Woods [19] was used to determine the threshold value *T* automatically. A pixel in a grayscale image with a grayscale value greater than *T* was assigned as the object pixel with binary value 1 (white) and otherwise as a background pixel with binary value 0 (black). Morphological openings with a 5 × 5 rectangle structural element and closings with a 3 × 3 rectangle structural element were used to remove the white spots in the background and the black spots in the object, respectively. Samples of the binary images are shown in Figure 5.

### 2.6. Volume Measurement

The steps for proposed volume measurement method are bounding box construction and generate 3D random points, Monte Carlo integration, and heuristic adjustment. The details of each step will be described in the following subsections.

#### 2.6.1. Bounding Box Construction

**Figure 6:**
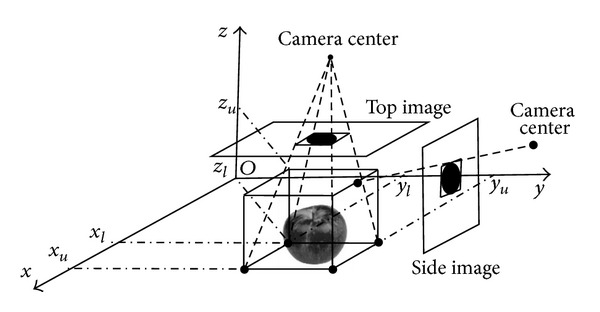
The illustration of bounding box construction.

The first step in the volume measurement using the Monte Carlo method was to determine the 3D bounding box, as the lower and upper bounds of the measured object in* x*,* y*, and* z* directions of real world coordinate system. The bounding box was constructed from binary images of the measured object and camera parameters, such that the measured object was completely contained in the box. The minimum bounding rectangles of the object were first created on the top view and on one of the side views of the binary images. The top bounding rectangle was reprojected onto the plane *z* = *z*_*l*_ located under the object and assumed as the lower bound in the* z* direction. The lower and upper bounds in the* x* and* y* directions were obtained from the reprojected top bounding rectangle by taking the minimum and maximum coordinates in the* x* and* y* directions. Suppose *x*_*l*_, *x*_*u*_, *y*_*l*_ and *y*_*u*_ are the lower and upper bounds in the* x* and* y* directions, respectively. To obtain the upper bound in the* z *direction (*z*_*u*_), the middle top point of the side bounding rectangle was reprojected onto one of the planes *x* = *x*_*l*_, *x* = *x*_*u*_, *y* = *y*_*l*_, or *y* = *y*_*u*_ closest to the point. The algorithm proposed by Siswantoro et al. [20] was used to reproject the points and bounding rectangles from the image coordinate system onto the real world coordinate system.  Figure 6 shows the illustration of the bounding box construction.

#### 2.6.2. Generate 3D Random Points

To perform Monte Carlo integrations, it is necessary to generate 3D random points in the bounding box *B* = {(*x*, *y*, *z*) | *x* ∈ [*x*_*l*_, *x*_*u*_], *y* ∈ [*y*_*l*_, *y*_*u*_], *z* ∈ [*z*_*l*_, *z*_*u*_]}. To degenerate a random point, three independent and identically distributed random variables uniformly distributed on [0, 1], *U*_1_, *U*_2_, *U*_3_, were generated *N* times. The random variables were then transformed into random variables uniformly distributed on [*x*_*l*_, *x*_*u*_], [*y*_*l*_, *y*_*u*_], and [*z*_*l*_, *z*_*u*_], respectively, using

(2) X=xl+(xu−xl)U1,Y=yl+(yu−yl)U2,Z=zl+(zu−zl)U3.

According to Walpole et al. [21], the 3D random variable (*X*, *Y*, *Z*) is uniformly distributed on the bounding box *B* = {(*x*, *y*, *z*) | *x* ∈ [*x*_*l*_, *x*_*u*_], *y* ∈ [*y*_*l*_, *y*_*u*_], *z* ∈ [*z*_*l*_, *z*_*u*_]}, with the probability density function in

(3) $$\begin{array}{c} p\left(x,y,z\right)=\{\begin{array}{cc} \frac{1}{V_{B}}, & \left(x,y,z\right)\in B\\ 0, & \text{otherwise}, \end{array} \end{array}$$

where *V*_*B*_ = (*x*_*u*_ − *x*_*l*_)(*y*_*u*_ − *y*_*l*_)(*z*_*u*_ − *z*_*l*_) is the volume of bounding box *B*.

#### 2.6.3. Monte Carlo Integration

Suppose the measured object is a closed bounded region *D* ⊂ *R* ^3^; then, the volume of the measured object can be obtained using the triple integral over *D*, as follows [12]:

(4) $$\begin{array}{c} V=\underset{D}{∭}dx\;dy\;dz. \end{array}$$

Because the measured object is contained in the bounding box *B*, the limit of integration in (4) can be extended to *B*, as in

(5) $$\begin{array}{c} V=\overset{z_{u}}{\underset{z_{l}}{\int }}\overset{y_{u}}{\underset{y_{l}}{\int }}\overset{x_{u}}{\underset{x_{l}}{\int }}G\left(x,y,z\right)dx\;dy\;dz, \end{array}$$

where

(6) $$\begin{array}{c} G\left(x,y,z\right)=\{\begin{array}{cc} 1, & \left(x,y,z\right)\in D\\ 0, & \left(x,y,z\right)\notin D. \end{array} \end{array}$$

Suppose *F*(*x*, *y*, *z*) = *V*_*B*_ *G*(*x*, *y*, *z*); then, the integral in (5) can be expressed as in

(7) $$\begin{array}{c} V=\overset{z_{u}}{\underset{z_{l}}{\int }}\overset{y_{u}}{\underset{y_{l}}{\int }}\overset{x_{u}}{\underset{x_{l}}{\int }}F\left(x,y,z\right)p\left(x,y,z\right)dx\;dy\;dz, \end{array}$$

where *p*(*x*, *y*, *z*) is a probability density function random variable uniformly distributed on *B*, as in (3). Let (*X*, *Y*, *Z*) be a random variable with a probability density function *p*(*x*, *y*, *z*); then, the right hand side of (7) is equal to the expected value of random variable *F*(*X*, *Y*, *Z*), as in

(8) $$\begin{array}{c} V=E\left(F\left(X,Y,Z\right)\right). \end{array}$$

Let random points (*x*_*i*_, *y*_*i*_, *z*_*i*_), *i* = 1,2,…, *N* be the independent realization of the random variable (*X*, *Y*, *Z*). Then, according to the Monte Carlo method [14], the expected value of *F*(*X*, *Y*, *Z*) could be approximated by the arithmetic mean of *F*(*x*_*i*_, *y*_*i*_, *z*_*i*_), as in

(9) $$\begin{array}{c} V\approx {\overset{-}{F}}_{N}=\frac{1}{N}\overset{N}{\underset{i=1}{\sum }}F\left(x_{i},y_{i},z_{i}\right). \end{array}$$

Because the integral in (4) was absolutely convergent, ${\overset{-}{F}}_{N}$ would be convergent in probability to *V*. This means that, for sufficiently large *N*, ${\overset{-}{F}}_{N}$ is very close to *V*.

To calculate the values of *F*(*x*_*i*_, *y*_*i*_, *z*_*i*_), the random points (*x*_*i*_, *y*_*i*_, *z*_*i*_) were projected onto all of the binary images of the object using the transformations in (10) and (11) as follows:

(10) $$\begin{array}{c} \left(\begin{array}{c} x_{cij}\\ y_{cij}\\ z_{cij} \end{array}\right)=R_{j}\left(\begin{array}{c} x_{i}\\ y_{i}\\ z_{i} \end{array}\right)+t_{j}.\\ \left(\begin{array}{c} x_{ij}\\ y_{ij}\\ 1 \end{array}\right)=\left(\begin{array}{ccc} f_{xj} & 0 & c_{xj}\\ 0 & f_{yj} & c_{yj}\\ 0 & 0 & 1 \end{array}\right)\left(\begin{array}{c} \frac{x_{cij}}{z_{cij}}\\ \frac{y_{cij}}{z_{cij}}\\ 1 \end{array}\right), \end{array}$$

where (*x*_*cij*_, *y*_*cij*_, *z*_*cij*_) is the coordinate of random point in *j*th camera coordinate system; **R**_*j*_, **t**_*j*_, *f*_*xj*_, *f*_*yj*_, *c*_*xj*_, *c*_*yj*_ are the extrinsic and intrinsic* j*th camera parameters; and (*x*_*ij*_, *y*_*ij*_) is the coordinate of random point projection on *j*th binary image, *j* = 1,2, 3,4, 5.

Suppose *f*_*j*_(*x*, *y*),  *j* = 1, 2, 3, 4, 5 are binary image values of a measured object from five different views; then

(11) $$\begin{array}{c} f_{j}\left(x,y\right)=\{\begin{array}{cc} 1, & \left(x,y\right)\;\text{is}\;\;\text{object}\;\;\text{pixel}\\ 0, & \left(x,y\right)\;\text{is}\;\;\text{background}\;\;\text{pixel} \end{array}j=1,2,3,4,5. \end{array}$$

Assume that if the projection of a random point falls in the object pixel for all binary images, then the original random point could be considered as a point in the object. Then, the value of *F*(*x*_*i*_, *y*_*i*_, *z*_*i*_) could be calculated using

(12) $$\begin{array}{c} F\left(x_{i},y_{i},z_{i}\right)=V_{B}\overset{5}{\underset{j=1}{\prod }}f_{j}\left(x_{ij},y_{ij}\right),\;i=1,2,\ldots ,N. \end{array}$$

By substituting (12) to (9), the volume of the measured object was approximated by

(13) $$\begin{array}{c} V\approx \frac{V_{B}}{N}\overset{N}{\underset{i=1}{\sum }}\overset{5}{\underset{j=1}{\prod }}f_{j}\left(x_{ij},y_{ij}\right). \end{array}$$

#### 2.6.4. Heuristic Adjustment

**Figure 7:**
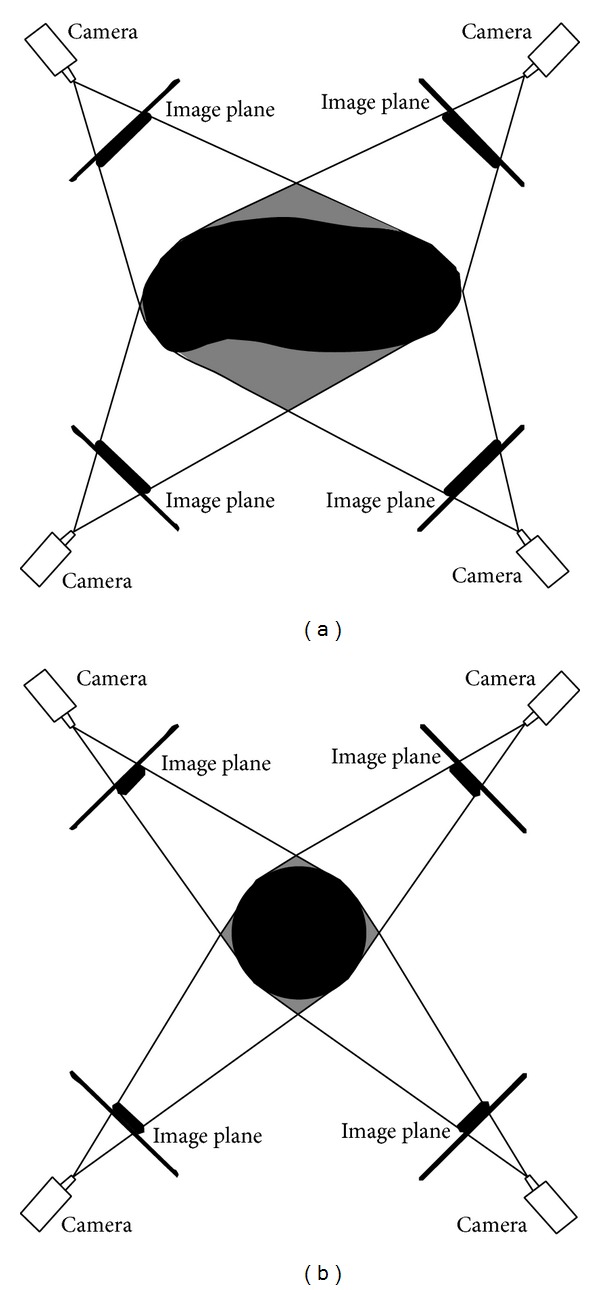
2D illustration of random point projection onto four binary images of object (left: mango, right: tomato). The projection of a random point in gray area (outside the object) will fall in object pixel for all binary images.

Because the computer vision system only used five cameras to capture the images of the measured object from five different views, it is possible that a random point located on the outside of the object was recognized as an object point, as illustrated in Figure 7. The projection of a random point located in the gray region of Figure 7 (the outside of object) will fall in the object pixel for all binary images, and this random point will be recognized as a point in the object. As a consequence, the results of the volume measurement will be greater than the actual volume. To obtain accurate measurement results, the proposed method employed a heuristic adjustment. As illustrated in Figure 7, the ratio between the actual volume (black area) and the volume measurement result (black and gray area) depended on the type of food product. Therefore, the amount of the adjustment also depended on the type of food product. To determine the amount of adjustment, five samples were chosen randomly from each type of food product. The volume of each sample was then measured using the proposed method before adjustment (*V*_*BA*_) and the water displacement method (*V*_*WD*_). The amount of adjustment (*K*) for each type of food product was calculated using

(14) $$\begin{array}{c} K=\text{mean}\left(\frac{V_{WD}}{V_{BA}}\right). \end{array}$$

Finally, the volume of the measured object was approximated using

(15) $$\begin{array}{c} V_{MC}=K\frac{V_{B}}{N}\overset{N}{\underset{i=1}{\sum }}\overset{5}{\underset{j=1}{\prod }}f_{j}\left(x_{ij},y_{ij}\right). \end{array}$$

### 2.7. Validation

The objects used to validate the proposed method were two balls (radius 3.67 cm and 3.68 cm, resp.) and 150 samples of food product with irregular shapes. The samples consisted of 50 apples, 50 mangoes, and 50 tomatoes. All samples were measured using the proposed method three times, and the means were calculated. For validation, the exact volumes of the balls (*V*_Ex_) and the experimental volumes of the food products (*V*_*WD*_) were calculated and measured, respectively. The exact volume of the balls was calculated using the following formula:

(16) $$\begin{array}{c} V_{\text{Ex}}=\frac{4}{3}\pi r^{3}, \end{array}$$

where *r* is the radius of ball. The experimental volume of the food product samples was measured using the water displacement method based on Archimedes' principle.

Absolute relative error (ARE) and coefficient of variation (CV) were used to measure the accuracy and precision of the proposed method. The ARE and CV were calculated using

(17) AREball=|VEx−VApp|VEx×100%

(18) AREfood  product=|VWD−VApp|VWD×100%

(19) CV=Std.  dev.VAppMeanVApp×100%,

where *V*_App_ is the volume approximation using computer vision. Furthermore, correlation coefficient and paired* t*-test were also used to show the goodness of the proposed method, statistically.

## 3. Results and Discussion

### 3.1. The Number of Random Points

Because the Monte Carlo method would give different results in different approximations, the ARE and CV were considered to specify the number of generated random points (*N*) used in volume measurement. The number of generated random points (*N*) was chosen such that the measurement results had a good accuracy and precision, as indicated by a small ARE and small CV.

The volume of a ball with radius 3.67 cm was measured 100 times using the proposed method with various numbers of random points, that is, 10^2^, 10^3^, 10^4^, 10^5^, 2 × 10^5^, 3 × 10^5^, 4 × 10^5^, 5 × 10^5^, 6 × 10^5^, 7 × 10^5^, 8 × 10^5^, 9 × 10^5^, and 10^6^. The ARE and CV were then calculated for each number of generated random points using (17) and (19), respectively, and the results are summarized in Table 1. Table 1 shows that the ARE and CV of ball volume tended asymptotically toward zero as the number of random points increased. For more than 10^5^ random points, the ARE and CV were less than 1%. Furthermore, for more than 5 × 10^5^ random points, increasing the number of random points did not significantly affect the decreased ARE and CV. Therefore, in this experiment, the number of random points was set to 8 × 10^5^ to obtain a good accuracy and precision.

### 3.2. Volume Measurement Results

The volume measurement results using the proposed method are summarized in Table 2. The summary of the volume measurement results using the Monte Carlo method before adjustment and using the water displacement method is also presented in this table. It can be observed in Table 2 that the mean volume measured using the proposed method was close to the mean volume measured by the water displacement method, with a mean ARE less than 1.00% for all objects. For all samples, the ARE was less than 3% for the 98% sample and less than 3.66% overall. It can be inferred that the result of the volume measurements from the proposed method had a high accuracy. In measuring the volume of balls, the proposed method produced a mean ARE 0.02%. In terms of food product samples, the mean ARE for apples (1.00%) was greater than that for mangos (0.97%) or tomatoes (0.82%). This may be explained by the fact that the apples used in the experiment had greater variation in shape compared with the other objects. The mean CV of ball volume measured using the proposed method was 0.10%. For food product samples, the proposed method produced almost same mean CV; these were 0.18% for apples, 0.16% for mangos, and 0.18% for tomatoes. For all samples, the CV was less than 0.5% for 98% of the samples and was less than 0.75% overall. This result shows that volume measurement using the proposed method provides a high level of precision. From Table 2, it can also be observed that the adjusted volume measurement based on the Monte Carlo method can reduce the ARE by more than 70%.

In this study, comparisons with space carving method [2] were also made to assess the accuracy of the proposed method. A summary of the volume measurement results using space carving method is also provided in Table 2. As shown in Table 2, space carving method produced a mean ARE greater than that of the proposed method or the Monte Carlo method before adjustment. Furthermore, a comparison of the total processing time between the proposed method and space carving method was performed in this study, as shown in Table 3. It can be observed from Table 3 that the proposed method is faster than the space carving method. This result shows that the proposed method is more accurate and lower in required computation time.

### 3.3. Statistical Analysis

**Figure 8:**
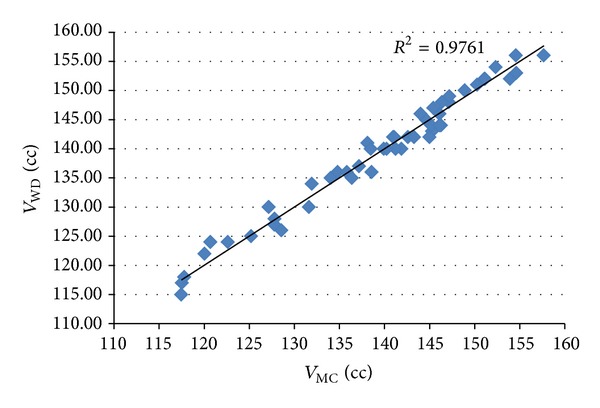
Correlation between the results of volume measurement using the proposed method and the water displacement method for apples.

**Figure 9:**
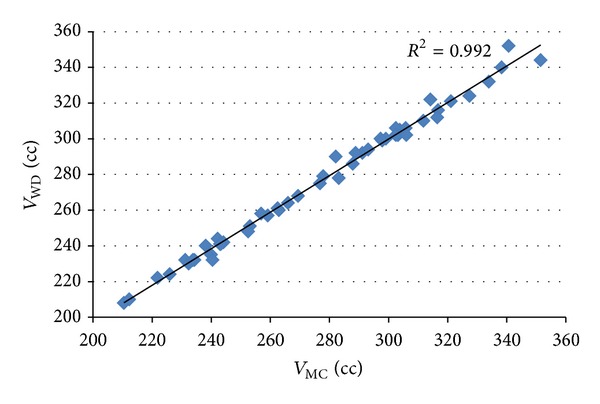
Correlation between the results of volume measurement using the proposed method and the water displacement method for mangos.

**Figure 10:**
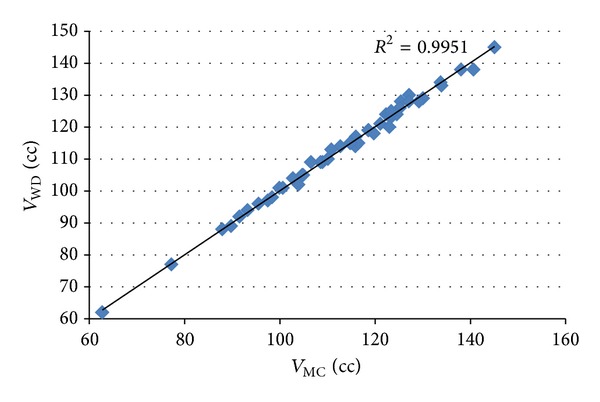
Correlation between the results of volume measurement using the proposed method and the water displacement method for tomatoes.

The correlation coefficient between the results of volume measurement using the proposed method and the water displacement method was used to measure the linear relationship between these two measurements. A strong correlation with the water displacement method was achieved by the proposed method for all types of sample, as shown in Figures 8, 9, and 10. The values of correlation coefficient (*R*) were 0.988, 0.996, and 0.998 for apples, mangos, and tomatoes, respectively. This shows that there is a good linear relationship between the results of volume measurement using the proposed method and the water displacement method for apples, mangos, and tomatoes. Because all *R* ^2^ were greater than 0.97, it can be said that more than 97% variation in the results of volume measurement using water displacement method can be accounted for by a linear relationship with the result of the volume measurement using proposed method.

Further statistical analysis for the difference between the mean volume measured using the proposed method and the water displacement method was the paired* t*-test. The paired* t*-test was used because the data came from same sample but were measured by two different methods. The normality for the different volumes was assessed by a normal plot using the* normplot* function in MATLAB. The paired* t*-test for the hypothesis that the difference between the mean volume measured using the proposed method and the water displacement method is zero was performed using the* t-*test function in MATLAB, with *α* = 0.05. The results of paired* t*-test are summarized in Table 4.

As shown in Table 4, the mean volume difference between the proposed method and the water displacement method was −0.0387, with a 95% confidence interval [−0.5042, 0.4268] for apples; 0.7011 with a 95% confidence interval [−0.2985, 1.7007] for mangos, and −0.1147 with a 95% confidence interval [−0.4684, 0.2391] for tomatoes. This shows that the volume measured using the proposed method was close to the volume measured using the water displacement method for all types of sample. Because all *P* values were greater than 0.05 for all types of sample, then it can be inferred that the mean volumes measured using the proposed method and the water displacement method are the same for all types of sample. This result is highly consistent with previous absolute relative error analyses.

## 4. Conclusion

In this paper, the method for nondestructive volume measurement of irregularly shaped food products based on the Monte Carlo method using a computer vision system is proposed. The proposed method employed five cameras to capture images of the food product from five different views. The captured images were processed to obtain binary images. Monte Carlo integration with heuristic adjustments was performed to measure the volume of food product based on the information extracted from the binary images. The proposed method was tested to measure the volume of food product with irregular shapes, such as apples, mangoes, and tomatoes. The experimental results show that the proposed method produced absolute relative error less than 3% for more than 98% of samples compared to the water displacement method and a coefficient of variation less than 0.5% for 98% of samples. For all samples, a high correlation (greater than 0.988) and no significant differences were achieved in the volume measurements using the proposed method and the water displacement method. Moreover, the proposed method is more accurate and faster than space carving method. Therefore, the proposed method could be used as a good alternative for measuring the volume of food product with irregular shapes. In future research, the application of the proposed method for volume measurement of food product moving in a conveyor belt should be investigated.

## Figures and Tables

**Table 1:** Mean ARE and CV of ball volume measured 100 times using the proposed method.

*N*	Mean ARE (%)	CV (%)
10^2^	12.4379	9.37355
10^3^	3.639812	3.001279
10^4^	1.177656	0.928611
10^5^	0.394737	0.320826
2 × 10^5^	0.255567	0.197934
3 × 10^5^	0.19354	0.154213
4 × 10^5^	0.169821	0.137531
5 × 10^5^	0.168033	0.127107
6 × 10^5^	0.160473	0.127936
7 × 10^5^	0.146126	0.11507
8 × 10^5^	0.135135	0.112324
9 × 10^5^	0.127982	0.103832
10^6^	0.103445	0.081879

**Table 2:** Volume measurement result using the proposed method, the water displacement method, and space carving method.

Object	Number of sample	Exact/water displacement	Monte Carlo method before adjustment			Proposed method			Space carving method	
		Mean *V*_Ex_/*V*_WD_ (cc)	Mean *V*_BA_ (cc)	Mean ARE (%)	Mean CV (%)	Mean *V*_MC_ (cc)	Mean ARE (%)	Mean CV (%)	Mean *V*_SC_ (cc)	Mean ARE (%)
Ball	2	207.90	229.43	10.35	0.10	207.86	0.02	0.10	231.93	11.56
Apple	50	139.32	157.78	3.80	0.18	139.28	1.00	0.18	146.95	5.48
Mango	50	276.26	313.66	13.61	0.16	276.96	0.97	0.16	319.19	15.63
Tomato	50	112.70	118.14	4.85	0.18	112.59	0.82	0.18	119.67	6.21

**Table 3:** Total processing time of volume measurement using the proposed method and space carving method.

	Proposed method	Space carving method
Average total processing time (s)	9.65	210.17

**Table 4:** The results of a paired *t*-test for the difference between the mean volumes measured using the proposed method and the water displacement method.

Sample	Paired differences		95% confidence interval for mean difference		*P* value
	Mean (cc)	Std. dev. (cc)	Lower	Upper	
Apple	−0.0387	1.6378	−0.5042	0.4268	0.8679
Mango	0.7011	3.5172	−0.2985	1.7007	0.165
Tomato	−0.1147	1.2447	−0.4684	0.2391	0.5178
